# Editorial: Waste to Energy: Biomass-Based Energy Systems

**DOI:** 10.3389/fbioe.2022.932981

**Published:** 2022-08-22

**Authors:** Hamid Mukhtar, Muhammad Waseem Mumtaz, Salvatore Massa

**Affiliations:** ^1^ Institute of Industrial Biotechnology, Government College University, Lahore, Pakistan; ^2^ Department of Chemistry, University of Gujrat, Gujrat, Pakistan; ^3^ Department of Agriculture, Food, Natural Resource and Engineering, University of Foggia, Foggia, Italy

**Keywords:** microbial fuel cell, anaerobic digestion, waste biomass applications, waste to bioenergy, biofuels (biodiesel and bioethanol)

Rapid urbanization and worldwide industrial development have prompted increasing energy demand thus imposing strain on global energy supply sources. To fulfill the higher energy demands, fossil fuels are being overexploited and their combustion is resulting in some of the most devastating damage to the environment such as greenhouse gas emissions, climate change, and air quality deterioration. So, there is a desperate need for safe, inexhaustible, sustainable, cost-effective, and eco-friendly processes for the production of bio-based energy. So, renewable energy resources are the best options to alleviate the impacts of climate change and environmental pollution and to assist in fulfilling future energy demands.

In recent years, the issue has been addressed by several researchers who succeeded in developing bio-based fuel sources i.e., bioethanol, biodiesel, microbial fuel cells, etc. However, fluctuation in cost and limited availability of these renewables remain ever-green concerns at the ecological, economic, and political levels. Waste to energy, specifically biomass-based, might be considered an excellent substitute in this regard. Plant biomass has been used as a significant fuel for heating and cooking purposes in different eras in almost all parts of the world, and even remains in current in use in some parts of theglobe. On the other hand, many developed countries are in the process of shifting toward biomass-based fuels and energy systems for transportation and electricity generation to avoid CO_2_ emissions through the burning of fossil fuels.

Biofuels incorporate bio-ethanol, biodiesel, biogas, and sustainable hydrocarbon fuels. These are broadly categorized as first to fourth generation biofuels depending on the feedstock and technology/method used for their production. First-generation biofuels, also called conventional biofuels, are those which are produced from food crops specifically grown on farmlands for the said purpose. Second-generation biofuels are those which are produced from different types of animal or plant non-food biomass while third-generation biofuels are those which are produced by using aquatic plant biomass such as algae. Fourth-generation biofuels are highly advanced and emerging types of biofuels which use novel technologies to specifically engineer plants or microorganisms for higher yields.

The two most frequently utilized biofuels are biodiesel and bioethanol. An extensive variety of feedstock is accessible for biofuel production including industrial wastes, agriculture residues, forest residues, and municipal solid waste. MSW (Municipal solid waste) is a significant contributor to the generation of renewable energy and to providing a sustainable climate. Considering the previously mentioned details, it has become a critical need of time for the development of inexpensive, eco-friendly, and sustainable processes for energy production. This requires cautious integration of different steps entailed in the production of biomass-based energy sources in a monetarily sound manner, for example, the choice of substrate (biomass or waste) for the production of renewable energy.

Keeping in view the above challenge, the current topic “*Waste to Energy: Biomass-Based Energy Systems*” was proposed to invite the researchers to share their valuable work in this area. This Research Topic has provided an overview of the production of biofuels from accessible agriculture residues and waste material, filling in as a feasible energy asset. In addition, the topic has also assisted in identifying and proposing novel ways to bridge the gaps in finding sustainable sources of renewable energy. Furthermore, the topic has brought forth recent advancements in the technologies and strategies for the generation of green and maintainable energy systems *via* the valorization of biomass and waste materials. The issue was expected to receive contributions in the areas of recent trends in the development of biorefineries, valorization of waste to bio-based energy, cellulolytic conversion of lignocellulosic to bio-alcohols, advances in the biogas production systems, advancements in pyrolysis process utilizing biomass for the development of bioenergy.

Miran et al., (present issue) has reported the modification of a traditional carbon felt (CF) electrode by iron oxide (Fe_3_O_4_) nanoparticles *via* facile dip-and-dry methods to enhance the efficiency of a microbial fuel cell (MFC) through the rapid electron transfer between microbes and electrodes. They utilized this SRB-enriched Fe_3_O_4_@CF anode to assist in enhancing MFC performance and efficient treatment of tannery wastewater. Through their findings, they concluded that a combination of the favorable properties of nanocomposites and efficient microbes for treating complex wastes can encourage new directions for renewable energy-related applications.

In the second article, Zainal et al., (present issue) assessed the potential of commercial Malaysian food waste (CMFW) as a sustainable bioenergy feedstock through biogas production. They evaluated the digestion process in continuous operation using a 6-L continuous stirred-tank reactor (CSTR). The authors recommended the CMFW as a sustainable feedstock for biogas production in Malaysia and suggested the authorities to introduce commercial scale CMFW AD as part of managing municipal solid waste issues in Malaysia and towards a circular economy approach.

Okedu et al., (present issue) provided an extensive review on strategies for using sustainable waste for efficient energy consumption in Oman. As the region of the Gulf Cooperation Council (GCC) member countries in the Middle East generates the highest quantity of municipal waste per capita compared to other countries globally, therefore, the authors have proposed waste to energy technologies envisioned in Oman for efficient waste management and increasing the power grid capability. Landfill leachate as an innovative feedstock to produce clean and green hydrogen gas using different photo-fermentation processes has been discussed. Furthermore, some challenges and opportunities in carrying out effective waste management in Oman were addressed.

Waste-to-energy technologies (WTE) have a number of advantages for developed as well as developing countries. Some of these advantages include waste management, reduction in the net greenhouse gas emissions, reduction in landfill expansion and energy generation, etc. A large number of countries have already adopted WTE strategies Briefly, it is imperative that adapting waste to energy technologies is to be achieved as soon as possible as it presents a significant contribution to fulfilling future requirements of energy demand. Besides, it will help in recycling and circular bioeconomy development as well. The article collection in the current topic encompasses from basic and traditional ways of utilizing waste biomass for energy generation to the advanced technologies such as microbial fuel cells for electricity generation utilizing wastewater. Hence, this collection will provide useful guidelines to the researchers working in the same area.

